# Coating Persistent Luminescence Nanoparticles With Hydrophilic Polymers for *in vivo* Imaging

**DOI:** 10.3389/fchem.2020.584114

**Published:** 2020-09-24

**Authors:** Jianhua Liu, Lenka Kotrchová, Thomas Lécuyer, Yohann Corvis, Johanne Seguin, Nathalie Mignet, Tomáš Etrych, Daniel Scherman, Eva Randárová, Cyrille Richard

**Affiliations:** ^1^Unité de Technologies Chimiques et Biologiques pour la Santé (UTCBS), CNRS UMR8258, Inserm U1267, Université de Paris, Paris, France; ^2^Institute of Macromolecular Chemistry, Czech Academy of Sciences, Prague, Czechia

**Keywords:** persistent luminescence, nanoparticles, surface coating, HPMA polymer, imaging, *in vivo*

## Abstract

Persistent luminescence nanoparticles (PLNPs) are innovative nanomaterials highly useful for bioimaging applications. Indeed, due to their particular optical properties, i.e., the ability to store the excitation energy before slowly releasing it for a prolonged period of time, they allow *in vivo* imaging without auto-fluorescence and with a high target to background ratio. However, as for most nanoparticles (NPs), without any special surface coating, they are rapidly opsonized and captured by the liver after systemic injection into small animals. To overcome this issue and prolong nanoparticle circulation in the bloodstream, a new stealth strategy was developed by covering their surface with poly(*N*-2-hydroxypropyl)methacrylamide (pHPMA), a highly hydrophilic polymer widely used in nanomedicine. Preliminary *in vivo* imaging results demonstrated the possibility of pHPMA as an alternative strategy to cover ZnGa_2_O_4_:Cr NPs to delay their capture by the liver, thereby providing a new perspective for the formulation of stealth NPs.

## Introduction

Persistent luminescence is the property of some materials which can remain luminescent for minute, sometimes hour after ceasing their excitation. This particular optical phenomenon is due to the existence of defects within the materials capable of storing excited electrons in traps before they recombine to emit photons (Brito et al., 2012). Since the first persistent luminescence phosphors (PLPs) discovered by Matsuzawa, a strontium aluminate (SrAl_2_O_4_:Eu^2+^,Dy^3+^) emitting green light (Matsuzawa et al., 1996), several bulk (micrometric size) PLPs emitting in the visual spectrum have been prepared, some of which are now commercially used as night-vision materials for various applications such as security and traffic signs, dials or luminous paints, owing to their strong and long persistent luminescence after excitation by the sun or ambient light^1^. Recently, our lab proposed the use of this physical phenomenon to develop new probes for *in vivo* imaging (Le Masne de Chermont et al., 2007). Most real-time optical *in vivo* imaging techniques, i.e., fluorescence imaging, use fluorescent probes that need to be excited at the same time as they are detected. However, the fluorescence emitted from the probe may be contaminated by the fluorescence from endogenous chromophores also excited during this process. This phenomenon, called auto-fluorescence, has the disadvantage of hindering the detection of the probe, especially when it is present in small quantities (Gao et al., 2004). We have shown that the *in vivo* application of persistent luminescence nanoparticles (PLNPs) containing appropriate luminescent ions (emitting photons in the near-infrared (NIR) range) can solve this problem (Maldiney et al., 2011a). Indeed, since PLNPs can store excitation energy before releasing it for a long time, it is possible to delay the acquisition of images, which permits *in vivo* imaging without auto-fluorescence, thus obtaining reliable information with very good sensitivity (Maldiney et al., 2014). Since this pioneering work, many research labs have utilized our approach and proposed various methods to prepare PLNPs of different sizes and compositions (Lécuyer et al., 2016) for imaging and more recently, for therapeutic applications, notably in photodynamic therapy (Liu et al., 2019; Tan et al., 2019). However, due to their size (~100 nm), these nanoparticles (NPs) are rapidly opsonized and captured by the liver once injected into the bloodstream (Maldiney, 2015). To avoid their fast clearance, it is necessary to chemically coat their surface with hydrophilic and unfolding molecules to prevent adsorption of proteins (such as opsonins) and to postpone their capture by the reticuloendothelial system (RES), especially Kupffer cells in the liver (Aggarwal et al., 2009).

Among the hydrophilic coating molecules with stealth unfolding properties preventing protein adsorption, several polymers based on polyethylene glycol (PEG) or poly(*N*-2-hydroxypropyl)methacrylamide (pHPMA) have been successfully used to coat the surface of various nanoparticles (Amoozgar and Yeo, 2012). Also, numerous nanosized systems based on pHPMA and its copolymers have been developed for targeted therapy and diagnostics of diverse malignancies, inflammatory diseases and other pathologies since they enable passive or active accumulation and controlled drug release at the target site (Chytil et al., 2017). The hydrophilic pHPMA is biocompatible and non-immunogenic and the versatile structure of pHPMA-based systems enables conjugation of numerous drug molecules, imaging agents or targeting moieties (Randárová et al., 2020), whereas the direct conjugation to PEG structure is limited by the absence of functional groups along PEG chain and several studies have proved the appearance of anti-PEG antibodies after *in vivo* application (Zhang et al., 2016). Moreover, the novel polymerization techniques, e.g., controlled reversible addition–fragmentation chain transfer (RAFT) polymerization, enable the synthesis of highly defined polymers with very low dispersity and high chain-end functionality (Šubr et al., 2013).

This study reports a novel strategy to chemically coat PLNPs made of ZnGa_2_O_4_ doped with Cr^3+^ (Cr being the ion responsible for emission in the NIR range) with pHPMA. The nanoassembly formed by the covalent grafting of pHPMA on PLNPs has been fully physicochemically characterized using different methods and preliminary evaluation of this coating has been performed *in vivo* on healthy mice. Moreover, the pHPMA-based coating was compared with another hydrophilic polymer coating, PEG-based, often used *in vivo* to obtain stealth nanoparticles.

## Materials and Methods

### Chemicals

Zinc nitrate hexahydrate (>99%) was purchased from Fluka. Gallium oxide (99.999%) and chromium (III) nitrate non-ahydrate (99.99%) were purchased from Alfa Aesar. (3-Aminopropyl)-triethoxysilane (99%) was obtained from Sigma-Aldrich. Dimethylformamide (>99.9%) was purchased from SDS. Alpha-methoxy-omega-N-hydroxysuccinimide poly(ethylene glycol) *M*_*W*_ 5.000 Dalton was bought from Iris Biotech GmbH. 1-Aminopropan-2-ol, methacryloyl chloride, tert-butanol (t-BuOH), dimethyl sulfoxide (DMSO), diethyl ether, ethyl acetate, acetone, methanol (MeOH), dimethylacetamide (DMA), dichloromethane (DCM), 2,2′azobis(isobutyronitrile) (AIBN), 4-cyano-4-(ethylthiocarbonothioylthio) pentanoic acid, 2-thiazoline-2-thiol, N-ethyl-N′-(3-dimethyl aminopropyl)carbodiimide hydrochloride were purchased from Merck-Sigma-Aldrich (Germany). Azo initiator V-70 was purchased from Wako Chemicals (Japan).

### Synthesis of ZGO

ZnGa_2_O_4_:Cr (ZGO) nanoparticles were prepared as previously reported (Maldiney et al., 2014). In brief, 8.94 mmol of gallium oxide was dissolved in 10 ml concentrated HNO_3_(35%), then the mixture sample was transferred to a Teflon-lined stainless steel autoclave and heated to 150°C overnight. After that, 0.04 mmol of Cr(NO_3_)_3_.9H_2_O and 8.97 mmol of Zn(NO_3_)_2_.6H_2_O were dissolved in 10 ml deionized water to form a transparent solution, which was mixed with the solution of Ga(NO_3_)_3_ under stirring. Then, ammonia solution was added drop by drop to adjust the pH to 7.5. The solution was stirred for 3 h at room temperature before transfer to a Teflon-lined stainless steel autoclave and heated to 120°C for 24 h. Finally, the resulting product was washed with water and ethanol several times, then dried at 60°C and sintered at 750°C for 5 h. Hydroxylation was performed by basic wet grinding of the powder (500 mg) for 15 min with a mortar and pestle in 2 mL of 5 mM HCl solution, and vigorous stirring overnight at room temperature. The hydroxylated ZGO obtained, or ZGO-OH NPs with a diameter of 40 nm were selected from the whole polydisperse colloidal suspension by centrifugation in a SANYO MSE Mistral 1,000 at 4,500 rpm for 10 min. ZGO-OH particles located in the supernatant (assessed by Dynamic Light Scattering) were collected and concentrated.

### Synthesis of p(HPMA)-TT

N-(2-hydroxypropyl)methacrylamide (HPMA) and thiazolidine-2-thione (TT) functional chain transfer agent (CTA) 2-cyano-5-oxo-5-(2-thioxo-1,3-thiazolidin-3-yl)pentan-2-yl ethyl carbontrithioate (TTc-TT) were synthesized as described previously (Kostka et al., 2020). Semitelechelic pHPMA with the TT end-groups p(HPMA)-TT was prepared by RAFT polymerisation of HPMA using 2,2′-azobis(4-methoxy-2,4-dimethylvaleronitrile) (V-70) as an initiator and TTc-TT as a CTA in the molar ratio of monomer:CTA:initiator 300:2:1. An example of the synthesis for p(HPMA)-TT is as follows: HPMA (2.0 g, 13.97 mmol) was dissolved in 17.0 mL of t-BuOH and mixed in a polymerisation ampule with TTc-TT (33.9 mg, 93.1 μmol) and initiator V-70 (14.4 mg, 46.6 μmol) dissolved in 3.0 mL of anhydrous DMA. The polymerisation mixture was bubbled with argon for 10 min and sealed. Polymerisation was conducted at 30°C for 72 h, then the polymer was precipitated in a mixture of acetone and diethyl ether (2:1), filtered and dried under a vacuum, yielding 1.5 g (75%) polymer. TTc groups from the polymer precursor were removed by the reaction with an excess of AIBN in DMA (80 °C, 3 h) (modified procedure from Perrier et al., 2005).

### ZGO Coating

Nanosized ZGO-OH particles were first converted to ZGO-NH_2_ NPs by adding 1 wt% of 3-aminopropyl-triethoxysilane (APTES) to a suspension of ZGO-OH NPs at 2.5 mg/mL in dimethylformamide (DMF). The reaction mixture was sonicated for the first 2 min using a Branson Ultrasonic Cleaner 1210 and vigorously stirred at room temperature for 6 h. Particles were then washed from unreacted APTES by three centrifugation and redispersion steps in DMF. ZGO-pHPMA and ZGO-PEG NPs were obtained by reacting ZGO-NH_2_ either with semitelechelic p(HPMA)-TT (10 μmol) or with NHS-PEG (10 μmol) at an NP concentration of 2.5 mg/mL in DMF. Then, NPs were centrifuged and washed 2 × with DMF and 2 × with water.

### Characterizations

#### Polymer Characterizations

The weight-average molecular weight M_w_, number-average molecular weight M_n_ and dispersity Ð* of polymer precursor and conjugates were measured using SEC on an HPLC Shimadzu system equipped with a SPD-M20A photodiode array detector (Shimadzu, Japan), differential refractometer (Optilab®rEX), and multiangle light scattering (DAWN HELLEOS II) detectors (both from Wyatt Technology Co., USA). The mobile phase for the TSKgel SuperSW3000 column was a mixture of methanol:sodium acetate buffer (CH_3_COONa:CH_3_COOH; pH = 6.5) (80: 20; v:v) and the flow rate was 0.3 mL min^−1^. The content of pHPMA chain end TT groups was determined spectrophotometrically in methanol using the extinction coefficient, ε_305_ = 10 500 L mol^−1^ cm^−1^.

#### Nanoparticle Characterizations

Dynamic light scattering (DLS) and zeta potential measurements were performed on a Malvern Zetasizer-Nano instrument equipped with a 4 mW He-Ne laser (633 nm). DLS measurements were recorded by diluting colloidal aqueous suspensions of ZGO at a concentration of 0.1 mg/mL in water. Colloidal stability tests were performed at a NP concentration of 2 mg/ml. Zeta potential measurements were performed in 20 mM NaCl solution. Transmission electron microscopy (TEM) was performed at 80 kV on JEOL JEM-100S using 5 μL of suspension dropped for 1 min. Thermogravimetric analysis (TGA) was performed using a TGA/DSC 1 from Mettler-Toledo (Greifensee, Switzerland) sensitive to 1 μg and calibrated beforehand with internal standard weights. Samples of approximately 2 mg were analyzed in alumina pans with a central hole of 1 mm diameter. All the experiments were performed at a 5 K.min^−1^ heating rate and under a dry airflow of 70 mL.min^−1^. Infrared spectra were recorded on an Affinity-1 (Shimadzu). Persistent luminescence decays were performed on an Optima camera (Biospace Lab, France). Acquisitions of the luminescence of nanoparticles were recorded for 5 min after a 2-min excitation under UV light (365 nm) or a LED lamp (5,000 ml) with a 515 nm filter.

#### *In vivo* Imaging

Eight-week-old Balb/cJRj female mice (18–22 g) were purchased from Janvier labs. *In vivo* experiments were approved by French Comité d'éthique en expérimentation animale N°034 and the French Ministry of Research (APAFIS#8519-20 16090514387844). The mice were randomly assigned to groups (*n* = 3) for experimental purposes. UV pre-excited NPs were injected retro-orbitally using insulin syringes (0.2 mL volume containing 10 mg/mL NP suspension in aqueous solution). At different time points after injection (1, 3, 7h), mice were irradiated for 2 min with LED, then imaged for 5 min with the Optima camera. Twenty-four hours after injection, animals were anesthetized with gaseous isoflurane and sacrificed by cervical dislocation. Liver, spleen, lungs, heart and kidneys were collected and placed on a black plate, irradiated with LED and *ex vivo* luminescence measurements were recorded.

## Results and Discussion

**Figure 1:**
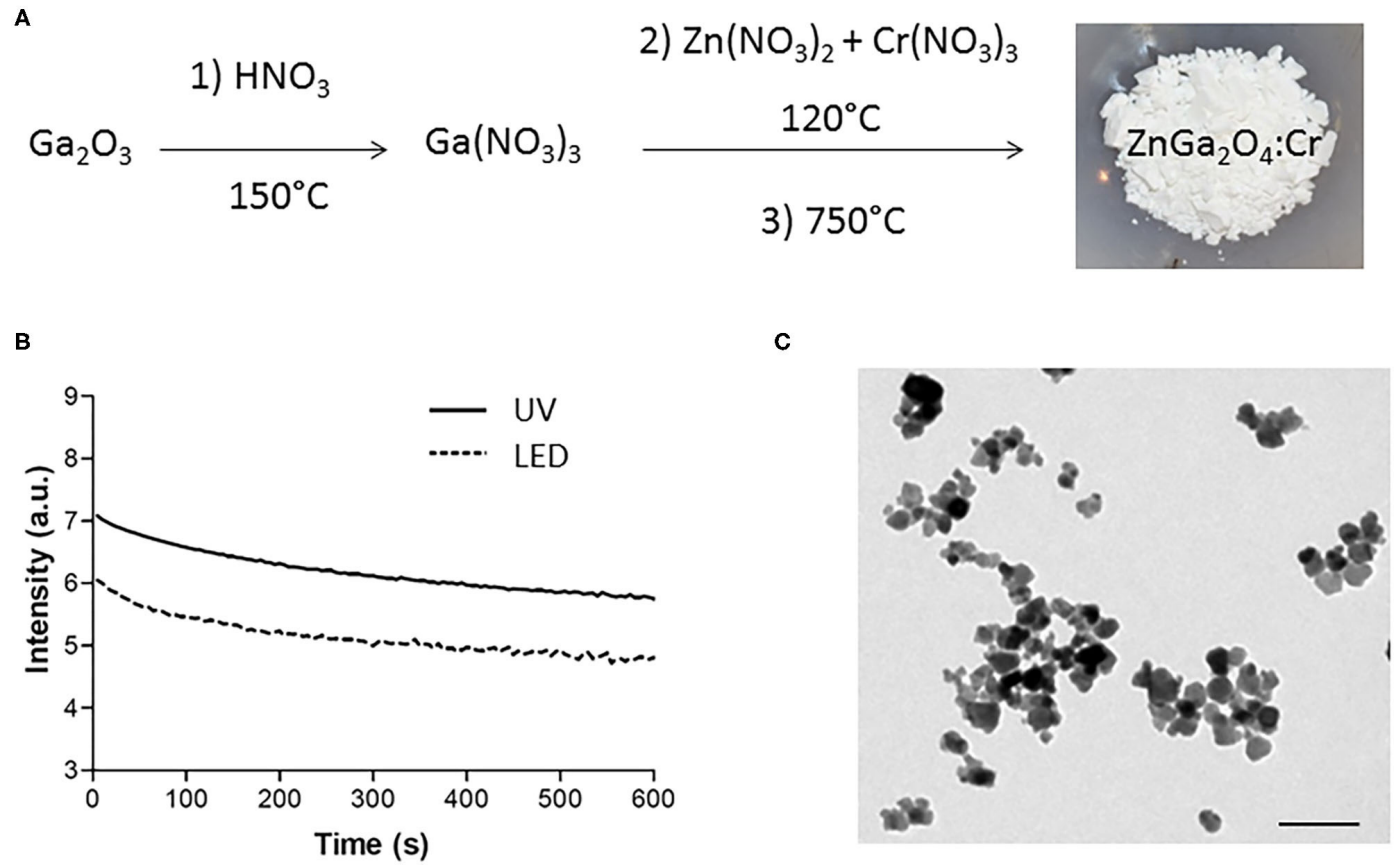
Synthesis and characterizations of ZnGa_2_O_4_:Cr (ZGO). **(A)** Three-step synthesis of ZGO. **(B)** Optical properties of ZGO powder after UV and visible excitation. **(C)** TEM of nanosized ZGO-OH (scale bar = 80 nm).

The chromium-doped zinc gallate NPs (ZnGa_1,995_Cr_0.005_O_4_, referred to as ZGO) were synthesized in a three-step method according to our previously published technique combining a hydrothermal treatment and 5-h calcination at 750°C (Figure 1A). The synthesized ZnGa_1,995_Cr_0.005_O_4_ could be excited with a wide range of wavelengths to give a NIR emission band at 697 nm, which was ascribed to the 2E → 4A2 transition in the distorted trivalent chromic ions in gallate (Supplementary Figure 1). Indeed, in agreement with previous results (Maldiney et al., 2014), the obtained powder can emit persistent luminescence signal for several minute after excitation in UV or visible light range (Figure 1B). As shown later in this work, emission of light over a long time after the end of excitation is highly useful for *in vivo* optical imaging without background. However, for this application, NPs should be recovered from the heterogeneous white powder. Nanosized hydroxylated ZGO (ZGO-OH) of approximately 40 nm in size were obtained by manual crushing of the powder, followed by acidic hydroxylation in HCl 5 mM overnight and extraction of nanoparticles by selective sedimentation, which was confirmed by TEM (Figure 1C).

Nevertheless, for *in vivo* applications, NPs surface functionalization is needed, since more than 3000 proteins can bind to the surfaces of non-functionalized NPs (Shemetov et al., 2012) in the bloodstream resulting in their rapid clearance by RES mainly present in liver and spleen, as already observed with ZGO-OH (Supplementary Figure 2). To overcome this issue, several strategies have been reported, such as the incorporation of PLNPs into liposomes (Chen et al., 2017) or by covalent grafting with PEG on the surface of the NPs (Maldiney et al., 2011b; Walkey et al., 2012). In this work, we propose another approach to achieve stealth NPs by coating ZGO with pHPMA, a biocompatible hydrophilic polymer already used for drug delivery in preclinical experiments (Šírová et al., 2017) and clinical trials (Kopeček and Kopečkova, 2010). This polymer (pHPMA-TT) was used to coat ZGO to evaluate the suitability of this coating for *in vivo* experiments and compared to non-functionalized ZGO-OH and PEGylated-ZGO.

**Figure 2:**
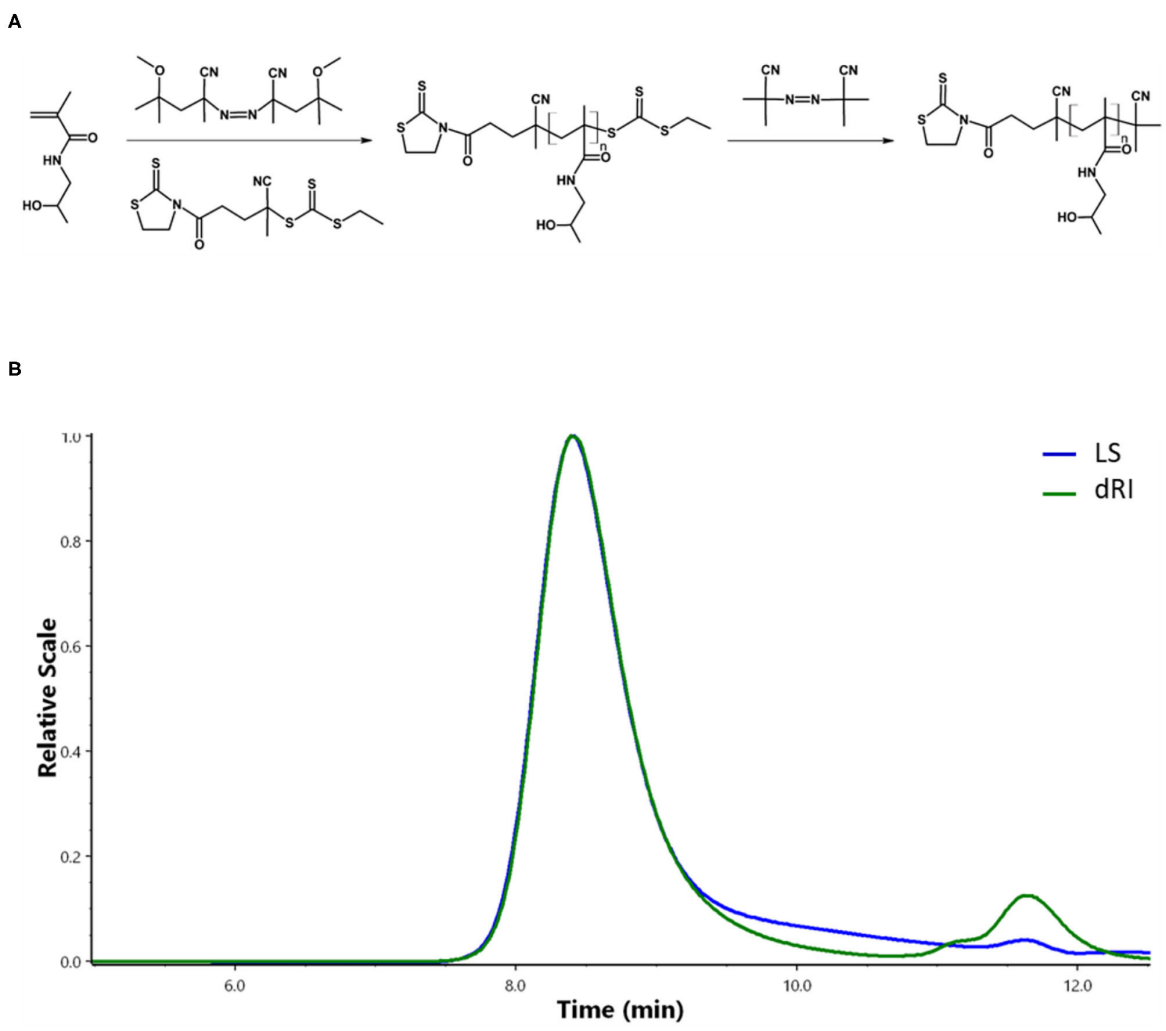
Synthesis and characterizations of pHPMA. **(A)** RAFT polymerization of HPMA. **(B)** Chromatogram of pHPMA-TT from SEC.

Semitelechelic linear polymer precursor p(HPMA)-TT intended for NP coating was synthesized by RAFT polymerisation using CTA TTc-TT and the azo initiator V-70 at 30 C providing polymer chains with sufficient functionality of the TT α-end group and very low distribution of molecular weights (Figure 2A).

The ratio monomer:CTA:initiator 300:2:1 was selected to obtain the polymer chains with the molecular weight around 20,000 g/mol since these pHPMA particles exhibit a comparable hydrodynamic radius to PEG with *M*_*w*_ = 5,000 g/mol selected as a control. The ω-TTc end-groups of p(HPMA)-TT were removed by a method (Perrier et al., 2005) that used an excess of AIBN in anhydrous DMA, thus preserving the content of the α-end TT groups in the polymers. The characteristics of p(HPMA)-TT are summarized in Figure 2B and Table 1.

**Table 1:** Physicochemical characterization of pHPMA precursor.

	**M_**w**_ (g/mol)^**a**^**	Ð*^a^	**R_**h**_ (nm)^**b**^**	**Content of TT groups (mol%)^**c**^**	**Functionality of TT groups^**d**^**
poly(HPMA)-TT	17 700	1.09	2.5 ± 0.3	0.65	0.70

a The molecular weights and Dispersity were determined by SEC using the TSKgel SuperAW3000 column and RI and MALS detection,

b the hydrodynamic radius was determined by DLS,

c the content of the TT end-chain groups was determined by spectrophotometry after the removal of the TTc groups,

d *polymer functionality of the TT end-groups (the ratio between the M_n_ value determined by SEC and the M_n_ value calculated from the content of the TT end-chain groups determined by spectrophotometry) after the removal of the TTc groups*.

**Figure 3:**
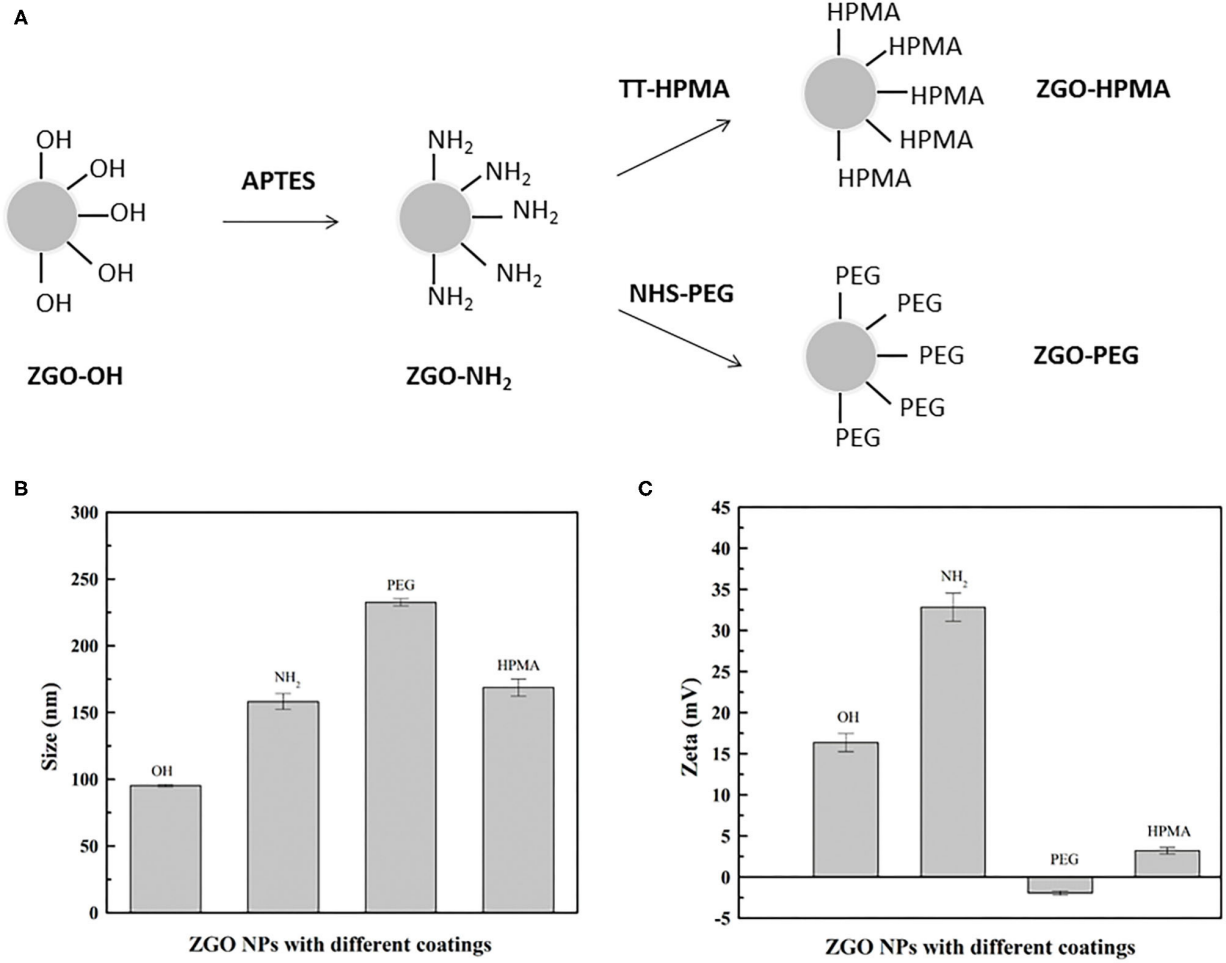
Coating of ZGO NPs. **(A)** Principle of ZGO coating with HPMA and PEG. **(B)** DLS of coated NPs. **(C)** Zeta potential of coated NPs.

Coating ZGO with both polymers was performed using a common functionalization strategy (Figure 3A). For this purpose, aminopropyltriethoxysilane (APTES) was first grafted on the ZGO-OH surface for 6 h in DMF (Figure 3A) (Maldiney, 2015). Then, after washing, the amine-covered nanoparticles (ZGO-NH_2_) were split into two vials, one reacting with a solution of pHPMA to form ZGO-pHPMA NPs, while the other one reacted with NHS-PEG in DMF for 16 h to form ZGO-PEG NPs as a control.

**Figure 4:**
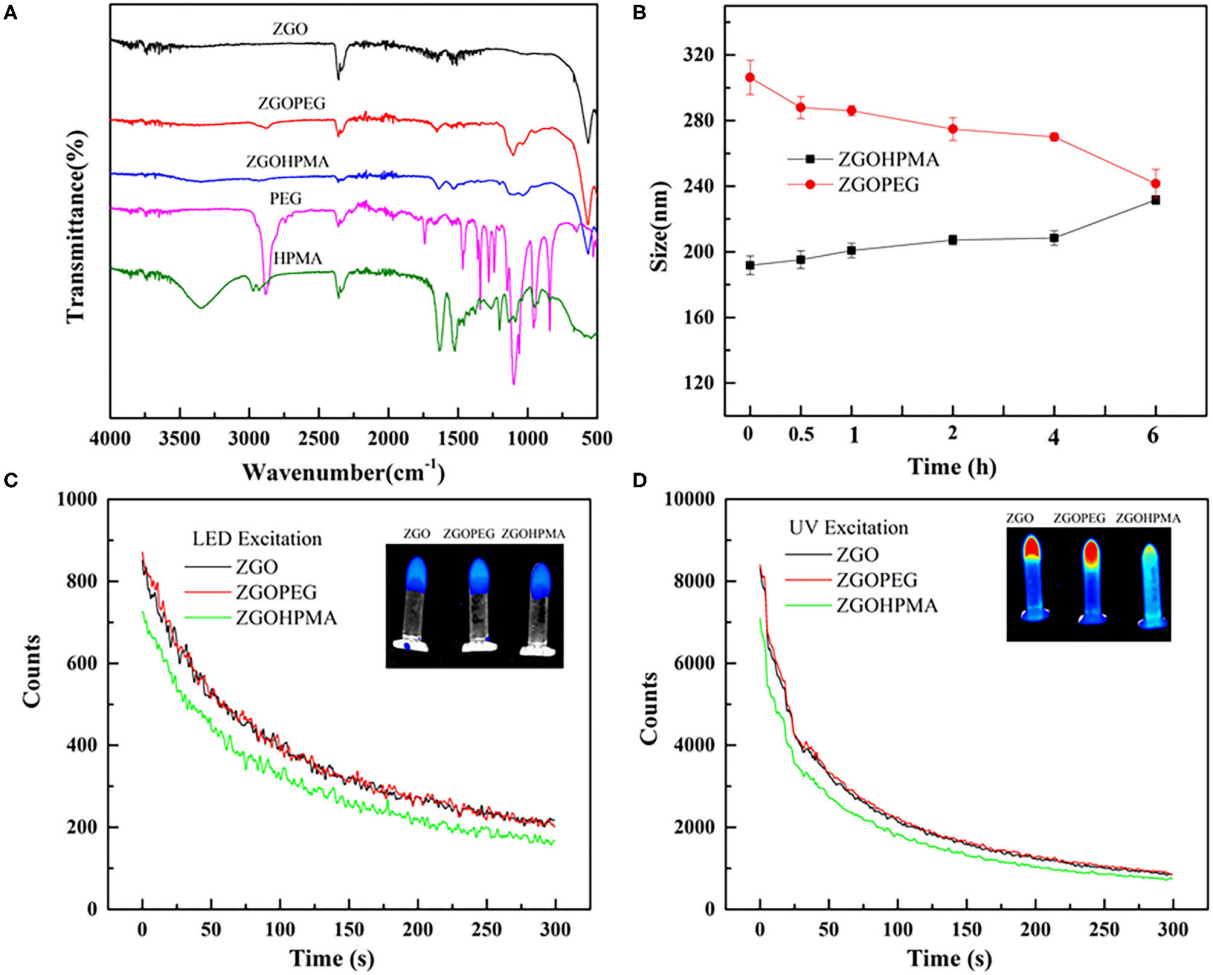
Physico-chemical characterizations of functionalized ZGO. **(A)** FT-IR spectra of polymers and ZGO-HPMA and ZGO-PEG. **(B)** Colloidal stability of ZGO-HPMA and ZGO-PEG in glucose. **(C)** Persistent luminescence decay after visible excitation. **(D)** Persistent luminescence decay after UV excitation.

Different physicochemical parameters were evaluated to confirm the coating of ZGO with pHPMA and PEG (Figures 3B,C, 4A and Supplementary Figure 3). After the reaction, the hydrodynamic diameter of ZGO-pHPMA nanoparticles increased from 90 to 170 nm. Regarding ZGO-PEG, their hydrodynamic diameter almost reached 230 nm after the 2-step functionalization process. The initial zeta potential of ZGO-OH nanoparticles was around 15 mV, 35 mV for ZGO-NH_2_. After functionalization with pHPMA, the zeta potential of ZGO-pHPMA was +2 mV, −1 mV after the addition of PEG (Figure 3C). Therefore, almost neutral NPs were obtained after coating ZGO with both neutral polymers, which is a prerequisite for the prevention of NP rapid uptake by RES. The presence of HPMA and PEG on ZGO were also confirmed by IR spectra, the characteristic peaks of each polymer being visible in Figure 4A. Indeed, the peaks at 2,875, 1,660, 1,075, and 925 cm^−1^ are characteristic of the PEG chain, while at 3,325, 2,950, 1,625, 1,525, and 1,100 cm^−1^ are typical of the HPMA moiety. Quantitative evaluation of the number of polymer chains grafted on ZGO was achieved by TGA. In the case of ZGO-PEG nanoparticles, the percentage weight loss stabilized at 4%, 5% for ZGO-pHPMA nanoparticles (Supplementary Figure 3). Starting from the molecular weight of each grafted polymer, we were able to quantify the concentration of pHPMA and PEG chains per mass unit of nanoparticles, around 4 nmol per mg of ZGO for pHPMA, close to 10 nmol/mg of nanoparticles for PEG. The different molecular weight and solution behavior of both polymers may play a role in the final coating density. Despite their hydrodynamic radii being comparable, the molecular weight of pHPMA was four times higher than of PEG. Also, pHPMA forms relatively expanded polymer coils in solution, while PEG chains are much more extended enabling better availability of end functional groups for the reaction with NP surface amino groups. Regarding the density of the coating, it was 0.8 molecule/nm^2^ for PEG and 0.3 molecule/nm^2^ for pHPMA. Indeed, a lower density coating for pHPMA explains the slightly higher zeta potential, which in this case, is influenced by the free amino groups on ZGO NPs after coating with pHPMA.

For *in vivo* use, nanoassemblies should not aggregate once dispersed into aqueous solution. The colloidal stability of both coated NPs was evaluated over 6 h after the dispersion at a concentration of 2 mg/ml in different aqueous solutions. As displayed on Supplementary Figure 4, when ZGO-pHPMA were dispersed in deionized water, their hydrodynamic diameter was constant during the first hour, slowly increasing to 220 nm over 6 h, whereas the ZGO-PEG NPs remained a constant size of approximately 180 nm. When dispersed in 5% glucose (Figure 4B), the hydrodynamic diameter of ZGO-pHPMA was stable with a Dh around 200 nm, whereas the Dh of ZGO-PEG slowly decreased from 300 nm (at T_0_) to 240 nm after 6 h.

To determine if the coating can influence the optical properties of ZGO NPs, persistent luminescence decay of colloidal suspensions of ZGO-pHPMA and ZGO-PEG were measured after UV and LED excitation and compared to non-coated ZGO-OH. As shown in Figure 4C, after a 2-min LED excitation, the characteristic luminescence decays of ZGO was observed after the excitation was stopped, with the intensity of light slowly decreasing over a 6-min period. The effect of the coating on the luminescence intensity was negligible, with only a slight decrease in the signal for ZGO-HPMA NPs compared to those of ZGO-OH or ZGO-PEG. A similar trend was observed after UV excitation (Figure 4D), while the obtained persistent luminescence intensity was about 10 times more powerful than after LED excitation, in line with our previously published results (Maldiney et al., 2014).

**Figure 5:**
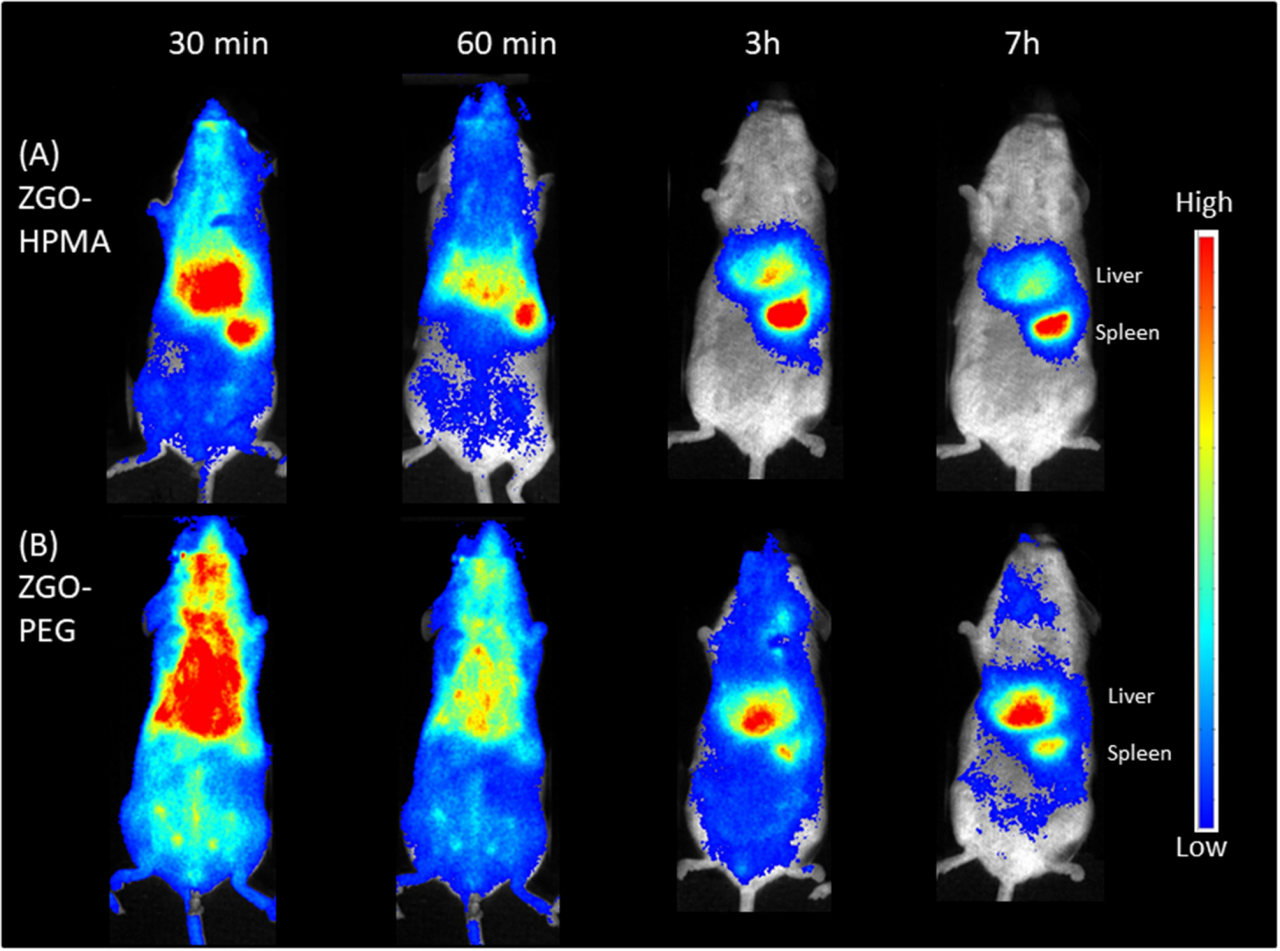
*In vivo* biodistribution of coated ZGO in healthy mice. **(A)** ZGO-HPMA. **(B)** ZGO-PEG.

Finally, preliminary *in vivo* imaging using ZGO coated with pHPMA was performed in healthy mice and compared to PEG-ZGO. For this purpose, suspensions of both NPs were pre-excited with a UV lamp for 2 min, then 200 μL (10 mg/mL) of each solution was injected retro-orbitally and the biodistribution of the NPs was followed over 7 h. After the injection of pHPMA-coated NPs, the luminescence signal was detectable throughout the whole animal body up to 60 min (Figure 5), proving the ability of pHPMA-coated NPs to circulate in the bloodstream, whereas the signal for the non-coated ZGO-OH was localized in the RES immediately after their application (Supplementary Figure 2), proving that the coating of ZGO with pHPMA is efficient and can successfully delay rapid NPs opsonization. For ZGO-PEG, the luminescence is also visible throughout the whole body, with less signal in the liver and spleen compared to ZGO-pHPMA. Three hours after injection, most ZGO-pHPMA resided in liver and spleen, whereas PEGylated-ZGO NPs were still circulating (Figure 5). The lower amount of HPMA linked to ZGO relative to PEG may be responsible for more capture by RES. In both cases, 7 h after injection, NPs were mainly localized in the liver and spleen.

**Figure 6:**
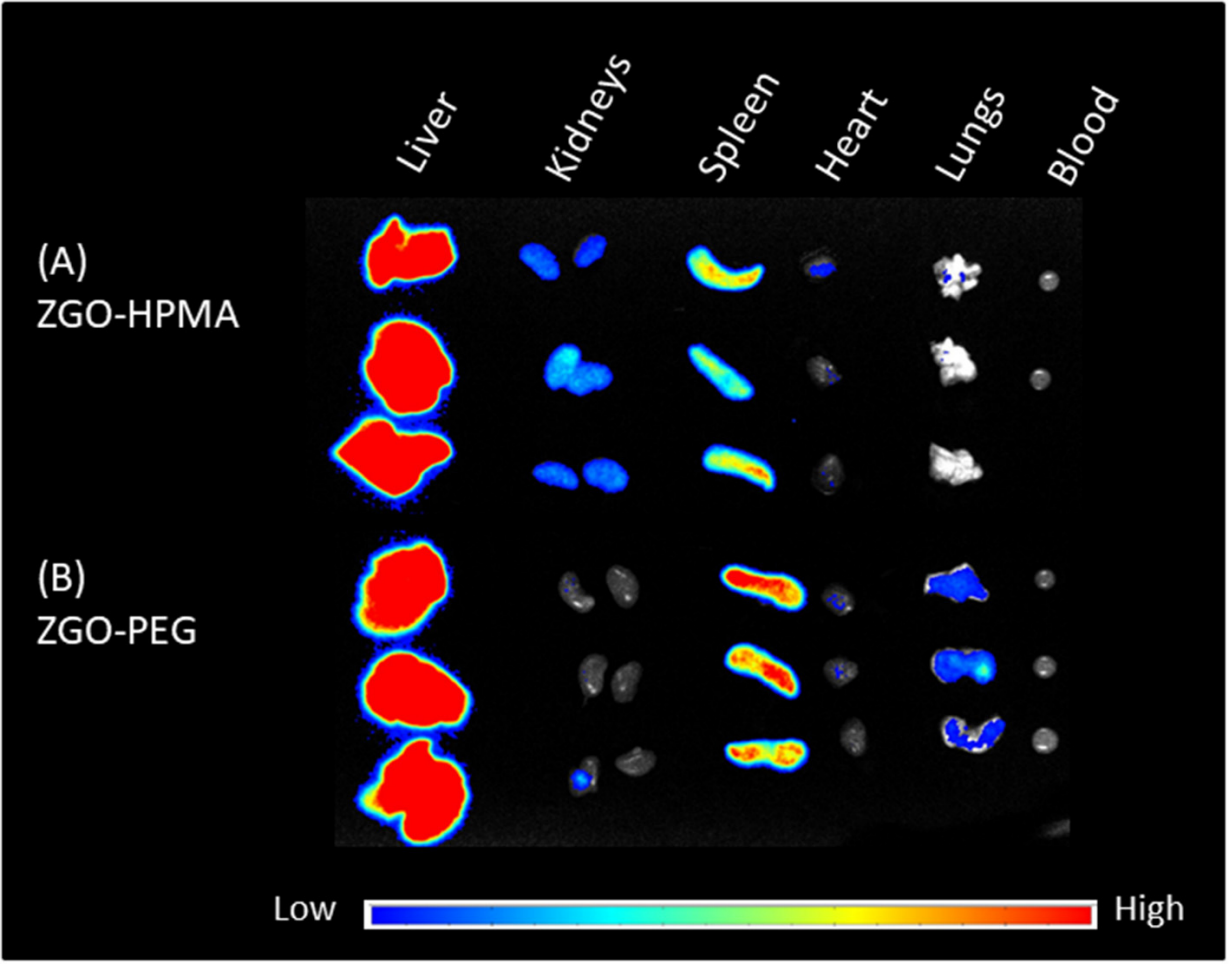
*Ex vivo* imaging of main organs and blood 24 h after injection. **(A)** ZGO-HPMA. **(B)** ZGO-PEG.

Twenty-four hours after injection, mice were sacrificed, and the main organs and blood were recovered and irradiated with LED for the *ex vivo* biodistribution study. For both coatings, the luminescence signal was mostly present in the liver and spleen (Figure 6). Nevertheless, there was a slight difference in organ biodistribution between coatings, since the *ex vivo* luminescence signal was found in the kidneys of ZGO-pHPMA injected mice, whereas for ZGO-PEG injected mice, there was no luminescence signal in the kidneys, a slight signal in the lungs and a stronger signal in the spleen. These *ex vivo* differences, a higher signal in the kidneys and lower signal in the lungs and spleen, constitute a real difference between HPMA- and PEG-coated NPs, hence an advantage and interest of using this newly developed coating strategy.

In summary, a new approach for coating ZGO nanoparticles was developed, which prolonged the circulation of the nanoparticles in the bloodstream. Since the residence time of ZGO-pHPMA in the blood was slightly shorter than for ZGO-PEG NPs, we assume that the pHPMA chain length and structure could be optimized to obtain a higher coating density, thus longer circulation time. It is hypothesized that either the comb-like pHPMA copolymer with multiple TT groups (layer-like coating) or shorter pHPMA copolymers grafted to ZGO nanoparticles in a higher density (brush-like coating), would bring new potential to the coating ability of ZGO NPs. Applied to smaller ZGO NPs (Teston et al., 2015) could also be of interest to modify the pharmacokinetic of injected NPs.

## Conclusion

We have reported the first use of pHPMA as an alternative strategy to coat ZGO NPs intended for *in vivo* imaging. ZGO NPs coated with the latter biocompatible polymer circulated in the bloodstream much longer than non-coated ZGO-OH. When compared to the PEG coating, it seems that the lower coating density of pHPMA chains slightly discriminate the pHPMA coating, thus the density or grafting approach should be optimized in future studies. Indeed, the pHPMA coating enabled more rapid elimination of coated NPs and decreased the long-term accumulation in organs such as spleen and lungs. Finally, the present results open new possibilities to improve ZGO coating for further applications in bioimaging and drug delivery, since the utilization of pHPMA-based coating enables the easy attachment of various drug molecules or targeting moieties.

## Data Availability Statement

The original contributions presented in the study are included in the article/Supplementary Material, further inquiries can be directed to the corresponding author/s.

## Ethics Statement

The animal study was reviewed and approved by French Comité d'éthique en expérimentation animale N°034 and by French Ministry of Research (APAFIS#8519-20 16090514387844).

## Author Contributions

JL: performed the experiments. LK: synthesized HPMA. TL: performed *in vivo* experiments. JS: injected nanoparticles to mice. YC: performed TGA. DS and NM: scientific discussion. TE: corrected the manuscript. ER: designed the experiments and corrected the manuscript. CR: designed the experiments and wrote the first version of the manuscript. All authors contributed to the article and approved the submitted version.

## Conflict of Interest

The authors declare that the research was conducted in the absence of any commercial or financial relationships that could be construed as a potential conflict of interest.

## Footnotes

^1^Available online at: https://www.nemoto.co.jp/nlm
