# Supplementary material for: Coating Persistent Luminescence Nanoparticles With Hydrophilic Polymers for in vivo Imaging
Source: Front Chem. 2020 Sep 24;8:584114. doi: 10.3389/fchem.2020.584114 (PMC7542242; doi:10.3389/fchem.2020.584114)
Supplement: Supplementary file 1 [file Data_Sheet_1.docx]

**Supplementary materials**

**Coating Persistent Luminescence Nanoparticles with Hydrophilic Polymers for *In Vivo* Imaging**

Jianhua Liu,^1^ Lenka Kotrchová,^2^ Thomas Lécuyer,^1^ Yohann Corvis,^1^ Johanne Seguin,^1^ Nathalie Mignet,^1^ Tomáš Etrych,^2^ Daniel Scherman,^1^ Eva Randarová^2^* and Cyrille Richard^1^*

Unité de Technologies Chimiques et Biologiques pour la Santé (UTCBS), CNRS UMR8258, Inserm U1267, Université de Paris, 75006 Paris, France

Institute of Macromolecular Chemistry, Czech Academy of Sciences, Heyrovského nám. 2, 162 06 Prague, Czech Republic

**
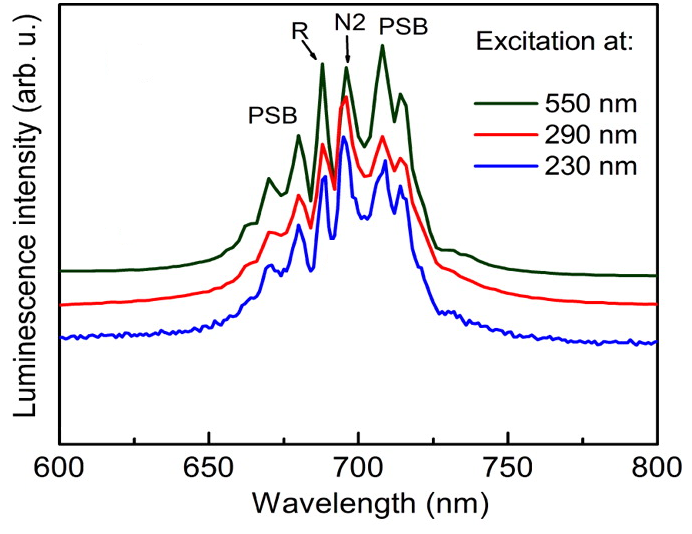
**

Fig S1. Emission spectrum of ZGO at different excitation wavelength.

Adapted from Chem Mater 2014, 26, 1365-1373.

Fig S2. *In vivo* imaging using non-coated ZGO-OH nanoparticles

Fig S3. Thermogravimetric analyses of functionalized ZGO-NH2 (blue), ZGO-PEG (green), and ZGO-HPMA (red). The weight loss percentages, i.e. 1.2%, 4.3%, and 5.2% have been determined between 220 and 550 °C due to -NH2, -PEG, and -HPMA thermal degradation, respectively.





Fig S4. Colloidal stability of functionalized ZGO in DI water
